# Exosomes from Placenta-Derived Mesenchymal Stem Cells Are Involved in Liver Regeneration in Hepatic Failure Induced by Bile Duct Ligation

**DOI:** 10.1155/2020/5485738

**Published:** 2020-10-09

**Authors:** Ji Hye Jun, Jae Yeon Kim, Jong Ho Choi, Ja-Yun Lim, Kyunggon Kim, Gi Jin Kim

**Affiliations:** ^1^Department of Biomedical Science, CHA University, Seongnam 13488, Republic of Korea; ^2^Department of Oral Pathology, College of Dentistry, Gangneung-Wonju National University, Gangneung 25457, Republic of Korea; ^3^Department of Integrated Biomedical and Life Sciences, College of Health Science, Korea University, Seoul 03722, Republic of Korea; ^4^Department of Convergence Medicine, School of Medicine, University of Ulsan and Asan Medical Center, Seoul 05505, Republic of Korea

## Abstract

Although the liver has a regenerative capacity, hepatic failure is a severe and irreversible chronic disease. Placenta-derived mesenchymal stem cells (PD-MSCs) have distinctive features, such as recycling of the placenta waste after birth, ease of accessibility, abundant cell numbers, and strong immunosuppressive properties. Previously, we reported that PD-MSCs can regenerate the liver in hepatic failure through antifibrotic and autophagic mechanisms. Many reports have investigated whether exosomes, which are formed by the budding of vesicular bodies and are emitted into the blood, from stem cells have therapeutic potential in various diseases. C-reactive protein (CRP) is produced in hepatocytes and secreted via vessels. Therefore, the objectives of this study were to compare the expression of CRP in exosomes of a hepatic failure rat model (bile duct ligation, BDL) and to evaluate the therapeutic effect by their correlation between CRP and angiogenesis depending on PD-MSC transplantation. The exosomes were analyzed in a BDL rat model with transplantation of PD-MSCs through LC-MS analysis and precipitation solution. The exosomes, CRP, and factors related to these molecules were evaluated and quantified in exosomes as well as investigated by real-time PCR, Western blot, and immunofluorescence (IF) *in vivo* and *in vitro*. CRP was present in exosomes from serum of a rat model and increased by PD-MSC transplantation. In the exosomes, CRP upregulated the factors related to the Wnt signaling pathway and angiogenesis in the BDL rat liver-transplanted PD-MSCs. Also, CRP regulated the Wnt pathway and vascularization in rat hepatocytes by interacting with endothelial cells. Therefore, our findings indicate that CRP in exosomes excreted by PD-MSCs functions in angiogenesis via the Wnt signaling pathway.

## 1. Introduction

Liver cirrhosis is the end stage of hepatic failure, which is irreversible [1]. Increased fibrosis, progressive hepatic vascular tone, and inflammation due to continuous hepatic damage are the main causes of mortality [2]. Although the liver has a powerful regeneration capacity, hepatic failure is triggered by several environmental factors, such as viral infection, chemical exposures, and chronic injuries [3]. In particular, cirrhosis is characterized by the spread of fibrosis, abnormal vascular architecture, and intrahepatic vascular shunts [4]. In cirrhosis, liver sinusoidal endothelial cells (LSECs) deform the vascular type, interfering with the exchange of molecules and resulting in blood flow resistance [5]. Therefore, vascular remodeling in liver tissues is a critical target for treating liver cirrhosis.

Mesenchymal stem cell (MSC) therapy is a promising strategy for hepatic diseases through overexpression of the hepatic growth factor (HGF) and reduction of collagen and matrix metalloproteinase (MMP) [6]. Additionally, the effect of umbilical cord-MSCs (UC-MSCs) on the attenuation of fibrosis via the TGFb-1 receptor through the TGFb-1/SMAD pathway was elucidated [7]. Human placenta-derived MSCs (PD-MSCs) have been categorized and assessed in stem cell therapy because they show proliferation, self-renewal, and low immunogenicity [8, 9]. Previously, we reported that chorionic plate-derived MSCs (CP-MSCs), which are one of several types of PD-MSCs, have the potential to differentiate into a variety of cell types including hepatocytes and osteocytes as well as the therapeutic effects of CP-MSCs in CCl_4_-injured rat liver through antifibrosis effects and autophagy [10, 11].

Exosomes are very small extracellular vesicles that are released from most cell types [12]. Biomarkers for exosomes include HSP70 and HSP90 as well as the tetraspanins CD9, CD63, CD81, and CD82 [13]. Exosomes are produced by the budding of multivesicular bodies and secreted from the cell membrane [14]. Exosomes contain many factors, such as proteins (cytoplasmic enzymes, adhesion and signal transduction molecules), microRNAs, and functional messenger RNAs [15]. Recently, exosomes have attracted interest as promising therapeutic targets [16]. Several reports have demonstrated that exosomes from stem cells have regenerative potential in hepatic failure [17]. In particular, bile-containing exosomes function as signaling nanovesicles and influence the regulatory mechanisms and proliferation of cholangiocytes via interactions with cilia [18]. Additionally, exosomes derived from hepatocytes transmit synthetic machinery, resulting in the proliferation of hepatocytes and regeneration after ischemic disease and hepatectomy [19]. In a previous report, we demonstrated that microRNA-125b in exosomes released from CP-MSCs repressed Hedgehog (Hg) signaling, which promotes hepatic fibrosis, indicating that microRNA-mediated Hh pathways assisted with liver regeneration by CP-MSCs [20]. However, which mechanisms and molecules from exosomes function in liver regeneration are unknown.

C-reactive protein (CRP), which is produced and released from the liver, is found in the vessel as a pentameric form. Circulating CRP (pentameric CRP, pCRP) breaks into monomers (monomeric CRP, mCRP), which have different characteristics than those of pCRP [21]. This protein is also well known to be an inflammatory protein that activates the complement cascade and macrophages [22]. CRP has been used as a diagnostic marker for a variety of inflammatory diseases because of its circulating properties [23]. Some studies have shown that mCRP enhances angiogenesis via PI3K signaling *in vitro*, interacting with Notch-3 [24]. Additionally, CRP was demonstrated to have high sensitivity and specificity as a diagnostic marker for cholangiocarcinoma and plays a role in improved prognosis [25]. However, it is not known whether CRP is related to regeneration in liver cirrhosis.

Therefore, the objective of this study was to analyze the profiling of exosomes released in a rat model with BDL by CP-MSC transplantation and demonstrate the therapeutic effect of CRP from the exosomes on a rat model with BDL after CP-MSC transplantation.

## 2. Materials and Methods

### 2.1. Animals

Seven-week-old male Sprague-Dawley rats were obtained (Orient Bio Inc., Seongnam, Korea) and maintained in an air-conditioned facility. The common bile duct was ligated under anesthesia with Avertin (2,2,2-tribromoethanol, Sigma-Aldrich). One week after the surgery, PKH67- (Sigma-Aldrich) labeled PD-MSCs (2 × 10^6^ cells, 8–10 passages) were injected intravenously through the tail vein in the transplanted group. The liver tissues and serum samples were harvested at 1, 2, 3, and 5 weeks from rats in all groups. All animal experimental processes were performed using a protocol consistent with the Institutional Review Board of CHA General Hospital, Seoul, Korea. The experimental protocols were approved by the Institutional Animal Care and Use Committee of CHA University, Seongnam, Korea (IACUC-180023).

### 2.2. Cell Isolation and Culture

All participants provided written and informed consent prior to sample collection. Placentas were collected from women who had no medical, obstetrical, or surgical complications and who delivered at term (37 gestational weeks). The collection of samples and their use for research purposes were approved by the IRB of CHA General Hospital, Seoul, Korea (IRB 07-18). PD-MSCs were harvested as described previously [26]. PD-MSCs were collected from the inner side of the chorionic membrane of the placenta. The cells scraped from the membrane were treated with 0.5% collagenase IV (Sigma-Aldrich) and cultured in Dulbecco's modified Eagle's medium/Ham's F-12 medium (DMEM/F12) (Invitrogen, Camarillo, CA) supplemented with 10% fetal bovine serum (FBS; Invitrogen), 1% penicillin/streptomycin (Pen-Strep, Gibco, Mulgrave, VIC, Australia), 25 ng/ml human fibroblast growth factor-4 (hFGF-4) (PeproTech, Inc., NJ), and 100 *μ*g/ml heparin (Sigma-Aldrich). Rat hepatocyte-like epithelial cells (WB-F344s) were cultured in *α*-MEM (Gibco) medium supplemented with 1% penicillin/streptomycin and 5% FBS (Gibco). Human umbilical vein endothelial cells (HUVECs) were cultured in EGM-2 SingleQuot (Lonza, Basel, Switzerland) medium at 37°C in a 5% CO_2_ incubator.

### 2.3. Cocultivation of WB-F344s, HUVECs, and PD-MSCs

WB-F344s (2 × 10^5^) and HUVECs (6 × 10^4^) were seeded onto cover glasses (Marlenfeld GmbH & Co., Huntington Beach, CA, USA) in a 100 mm dish culture plate (Corning, Corning, NY, USA). After 3 hours, WB-F344 and HUVECs were exposed to 100 *μ*M lithocholic acid (LCA) for 24 hours. The next day, PD-MSCs (6 × 10^4^) were cocultured in Transwell inserts (3 *μ*m pore).

### 2.4. Treatment with Recombinant CRP in WB-F344s, HUVECs, and PD-MSCs

CRP was upregulated by treatment of recombinant CRP (R&D systems, Minneapolis, MN, USA). The recombinant CRP reagents were diluted with complement medium and added to each well. The final concentration of recombinant CRP was 1 *μ*g/ml. After incubation of recombinant CRP for 24hours, the expression level of CRP was determined by immunofluorescence.

### 2.5. Transfection with Small Interfering RNA in WB-F344s, HUVECs, and PD-MSCs

Expression of CRP was silenced by transfection with small interfering RNA (siRNA) specific for CRP. siRNAs for human (INV-12990-01) and rat (INV-13300-01) CRP were purchased from Invitrogen. For siRNA transfection, Lipofectamine 2000 (Invitrogen) reagent was used according to the manufacturer's instructions. Lipofectamine 2000 reagent was incubated with Opti-MEM (Gibco) for 5 minutes, and respective siRNAs were then added to the mixtures for 20 minutes at room temperature. After incubation, the mixtures were diluted with serum-free medium and added to each well. The final concentration of siRNAs was 20 nM. After incubation of siRNAs for 24 hours, the cells were cultured in growth medium, and the expression levels of CRP and GAPDH were then determined by qRT-PCR analysis.

### 2.6. Magnetic Cell Sorting (MACS)

To sort the cultured WB-F344s and HUVECs, we performed MACS. First, harvested WB-F344s and HUVECs were incubated with biotin-conjugated anti-CD31 (Miltenyi Biotec Inc., Auburn, CA, USA) for 10 min. Antibiotin microbeads (Miltenyi Biotec Inc.) were reacted with collected cells for 15 min. Conjugated cells were sorted using MidiMACS Starting Kits (Miltenyi Biotec Inc.).

### 2.7. SDS-PAGE Separation

Equal amounts of serum from individual animals were pooled (*n* = 5). To isolate exosomes from the rat serum, we used the ExoQuick Exosome Precipitation Solution (System Biosciences, SBI, Palo Alto, CA, USA). The solution for exosome precipitation was used to analyze the proteome. Each exosome sample from plasma was mixed with 5x SDS loading buffer and boiled for 10 min at 80°C on a heat block. After centrifugation, each protein sample was subjected to 12% SDS-PAGE. After visualization using Coomassie brilliant blue, the gel of each lane was cut into 8 fractions and then cut into small pieces for in-gel digestion.

### 2.8. Enzymatic In-Gel Digestion

For digestion, gel pieces were washed with distilled water 3 times to remove SDS and dehydrated using 100% acetonitrile. Protein reduction using 10 mM DTT in 50 mM NH_4_HCO_3_ for 45 min at 56°C was performed. After the samples were washed with 100% acetonitrile, alkylation of cysteines was performed with 55 mM iodoacetamide in 50 mM NH_4_HCO_3_ for 30 min in the dark. Finally, each dehydrated gel piece was treated with 12.5 ng/*μ*l sequencing-grade modified trypsin (Promega, Madison, WI, USA) in a 50 mM NH_4_HCO_3_ buffer (pH 7.8) at 37°C overnight. Following digestion, tryptic peptides were extracted with 5% formic acid in 50% ACN solution at room temperature for 20 min. The supernatants were collected and dried with a SpeedVac. Resuspended samples in 0.1% formic acid were purified and concentrated using C18 ZipTips (Millipore, MA) before mass spectrometry (MS) analysis.

### 2.9. Nano-LC-ESI-MS/MS Analysis

Peptide separation was performed using a Dionex UltiMate 3000 RSLCnano system (Thermo Fisher Scientific). Tryptic peptides from the bead column were reconstituted using 0.1% formic acid and separated on a 50 cm EASY-Spray column with a 75 *μ*m inner diameter packed with 2 *μ*m C18 resin (Thermo Scientific, USA) over 120 min (300 nl/min) using a 0 to 45% acetonitrile gradient in 0.1% formic acid at 50°C. The LC was coupled to a Q Exactive mass spectrometer with a nano-ESI source. Mass spectra were acquired in a data-dependent mode with an automatic switch between a full scan and 5 data-dependent MS/MS scans. The target value for the full scan MS spectra was 3,000,000 with a maximum injection time of 120 ms and a resolution of 70,000 at *m*/*z* 400. The ion target value for MS/MS was set to 1,000,000 with a maximum injection time of 120 ms and a resolution of 17,500 at *m*/*z* 400. Dynamic exclusion of repeated peptides was applied for 20 s.

### 2.10. Database Searching

The resulting raw files were processed using MaxQuant (version 1.5.2.8) for identification with the database of *Homo sapiens* (organism ID: 9606, 71567 entries, UniProt). The search parameters were set as default, including cysteine carbamidomethylation as a fixed modification, N-terminal acetylation, methionine oxidation phosphoserine, phosphothreonine, and phosphotyrosine as variable modifications and diglycine modification at lysine residues with 2 missed cleavages. Peptide identifications were based on a search with an initial mass deviation of the precursor ion of up to 10 ppm, and the allowed fragment mass deviation was set to 20 ppm.

### 2.11. Postdata Analysis

The resulting files from MaxQuant were processed using Scaffold software (version 4.4.1.1, Proteome Software Inc., USA) for statistical analysis and visualization. Gene ontology analysis using UniProt accession numbers was performed using the Database for Annotation, Visualization and Integrated Discovery (DAVID) v6.8 (https://david.ncifcrf.gov/), and Venn diagram analysis was performed using the jvenn website (http://jvenn.toulouse.inra.fr).

### 2.12. Quantitative Real-Time PCR

Total RNA was isolated from rat liver tissues and cells using TRIzol (Invitrogen). Reverse transcription was performed with 500 ng of total RNA and Superscript III reverse transcriptase (Invitrogen). cDNA was amplified by PCR. Real-time PCR was performed using SYBR Ex Taq (Roche, Pleasanton, CA, USA) and an Exicycler™ 96 quantitative thermal block (Bioneer, San Francisco, CA, USA). The PCR reaction conditions were as follows: denaturation at 95°C for 15 min and 20 s, followed by 40 cycles of 95°C for 30 s and annealing at 52~60°C for 40 s. Extension at 70°C for 15 min and a final extension at 72°C for 7 min were performed. Gene expression was normalized to that of GAPDH. The sequences of the primers were as follows: ratCRP forward 5′-GCT TTT GGT CAT GAA GAC ATG TC-3′, ratCRP reverse 5′-TCA CAT CAG CGT GGG CAT AG-3′, rat Axin2 forward 5′-AAA CCT ATG CCT GTC TCC TC-3′, rat Axin2 forward 5′-ATC CAC ACA TTT CTC CCT CT-3′, rat VEGF forward 5′-ACG GAC AGA CAG ACA GAC AC-3′, rat VEGF reverse 5′-CTT CTG GGC TCT TCTTCT CTC TC-3′, rat albumin forward 5′-GCC CCA GAA CTC CTT TAC TA-3′, rat albumin reverse 5′-AAT CTC TGC ATA CTG GAG CA-3′, rat GAPDH forward 5′-TCC CTC AAG ATT GTC AGC AA-3′, rat GAPDH reverse 5′-AGA TCC ACA ACG GAT ACA TT-3′, human CRP forward 5′-TCG TAT GCC ACC AAG AGA CAA GAC A-3′, human CRP reverse 5′-AAC ACT TCG CCT TGC ACT TCA TAC T-3′, human Axin2 forward 5′-TCC CCA CCT TGA ATG AAG AA-3′, human Axin2 reverse 5′-TGG TGG CTG GTG CAA AGA-3′, human VEGF forward 5′-GCC TTG CCT TGC TGC TCT AC-3′, human VEGF reverse 5′-ACA TCC ATG AAC TTC ACC ACT TCG-3′, human albumin forward 5′-TGA GAA AAC GCC AGT AAG TGA C-3′, human albumin reverse 5′-TGC GAA ATC ATC CAT AAC AGC-3′, human GAPDH forward 5′-GCA CCG TCA AGG CTG AGA AC-3′, and human GAPDH reverse 5′-GTG GTG AAG ACG CCA GTG GA-3′.

### 2.13. Western Blot Analysis

Homogenized rat liver tissues and WB-F344s were lysed in RIPA buffer (Sigma-Aldrich) supplemented with a protease inhibitor cocktail (Roche) and phosphatase inhibitor (Sigma-Aldrich). A total of 45 *μ*g protein extracts were separated in gels of sodium dodecyl sulfate polyacrylamide gel electrophoresis (SDS-PAGE). The separated proteins were transferred onto PVDF membranes (Bio-Rad, Hercules, CA, USA). The membrane was incubated with rabbit anti-CRP (1 : 1,000, Abcam Inc., Cambridge, MA, USA), rabbit anti-Wnt3a (1 : 1,000, Abcam Inc.), mouse anti-Wnt4 (1 : 1,000, CUSABIO, China), rabbit anti-Wnt5a (1 : 1,000, Invitrogen), mouse anti-HSP70 (1 : 1,000, StressMarq Biosciences, Waltham, MA, USA), mouse anti-VEGF (1 : 1,000, R&D Systems), goat anti-VEGFR2 (1 : 500, R&D Systems), rabbit anti-phospho-LRP6 (1 : 500, Cell Signaling, Danvers, MA, USA), rabbit anti-*β*-catenin (active form; 1 : 500, Cell Signaling), rabbit anti-GSK3 (1 : 1,000, Cell Signaling), rabbit anti-*β*-catenin (active form; 1 : 500, Cell Signaling), rabbit anti-albumin (1 : 1,000, Novus, St. Louis, Missouri, USA), mouse anti-actin (1 : 3,000, Sigma-Aldrich), and mouse anti-tubulin (1 : 3,000, Abcam, Inc.) at 4°C overnight. The membrane was incubated with horseradish peroxidase- (HRP-) conjugated secondary anti-mouse IgG (1 : 5000, Cell Signaling), anti-rabbit IgG (1 : 10,000, Cell Signaling), or anti-goat IgG (1 : 5000, Santa Cruz Biotechnology, Dallas, Texas, USA) for 1 hour at room temperature. The bands were detected using the Clarity Western ECL kit (Bio-Rad).

### 2.14. Enzyme-Linked Immunosorbent Assay (ELISA)

The concentration of CRP was determined by ELISAs. Equal amounts of serum from individual animals were pooled (*n* = 5). All reactions were performed in triplicate. The concentration of CRP was measured using a rat C-reactive protein ELISA kit (Invitrogen) according to the manufacturer's instructions.

### 2.15. Immunofluorescence

For analysis of the localization of *β*-catenin, BrdU, and CRP in WB-F344s, the WB-F344s cocultivated with PD-MSCs were fixed with 4% paraformaldehyde (PFA) and permeabilized with 0.25% Triton X-100 and 3% H_2_O_2_. The cells were incubated with blocking solution (Dako, Carpinteria, CA, USA) at room temperature for 40 minutes and rabbit anti-*β*-catenin (1 : 80, Cell Signaling), rabbit anti-CRP (1 : 200, Santa Cruz Biotechnology), and mouse anti-BrdU (1 : 100, Cell Signaling) at 4°C overnight. After the reaction, the cells were incubated with Alexa 568 and 488- (1 : 100, Invitrogen) conjugated secondary antibody at room temperature for 1 hour. The slides were stained with 4′,6-diamidino-2-phenylindole (DAPI) for counterstaining. The images were observed with a fluorescence microscope EVOS (Thermo Fisher Scientific, Waltham, MA, USA) or confocal microscope (LSM 700). All experiments were performed in triplicate.

### 2.16. Statistical Analysis

All experiments were conducted in duplicate or triplicate. Data are expressed as mean ± standard deviation. Student's *t*-tests were performed for group-wise comparisons, and *p* < 0.05 was considered statistically significant. Statistical analyses were performed using PASW version 22.0 (SPSS Inc., Chicago, IL, USA).

## 3. Results

### 3.1. Engraftment and Tracing of PD-MSC in BDL Rat Liver

To demonstrate the engraftment of PD-MSC into the injured liver tissues, we labeled PD-MSCs with PKH67 fluorescence (green) labeling dye. As shown in the Supplement Figures, the green signal was not detected in the liver of tissues of the nontransplantation (NTX) group; otherwise, the PKH67 positive signal was strongly detected in the liver tissues of the transplantation (TTX) group at 1 week (wk). The signals were gradually decreased in 2, 3, and 5 wks in TTX groups (^∗^*p* value ≤ 0.05) (Supplement Figures 1(a) and 1(c)). Also, the expression of human-specific Alu sequences in rat liver tissues was significantly increased in the TTX group compared to the NTX group (^∗^*p* value ≤ 0.05) (Supplement Figure 1(b)). These data mean that transplanted PD-MSCs are engrafted in rat liver tissues until 5 wks although their populations are decreasing time-dependently.

### 3.2. The Analysis of Exosomes from Bile Duct Ligation Rat Liver and the Expression of CRP

**Figure 1:**
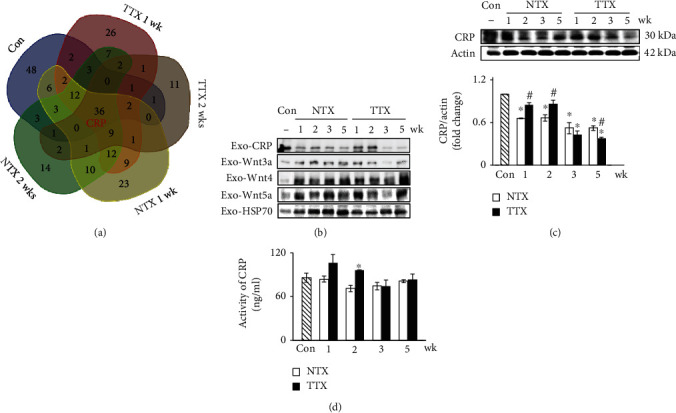
Increased CRP and Wnt ligands from exosomes by PD-MSC transplantation in bile duct-ligated rats. Exosomes were precipitated from BDL rat serum by LC-mass spectrometry (a). The expression of CRP and Wnt ligands from exosomes was normalized by HSP70 in rat serum after PD-MSC transplantation (b). The protein levels of CRP were determined by Western blot (c) and ELISA (d) after PD-MSC transplantation (*n* = 3 per group, ^∗^ Con and ^#^NTX vs. *p* < 0.05). Data were represented as the mean ± SD. CRP: C-reactive protein; HSP70: heat-shock protein 70; Con: control group; NTX: PD-MSC nontransplanted group; TTX: PD-MSC transplanted group; wk: week.

To verify the factors included in exosomes from the BDL rat liver, we isolated the exosomes from the rat serum, and the proteomes of the exosomes were analyzed using mass spectrometry (Figure 1(a)). As shown Figure 1(a), the CRP was detected in exosomes from rat serum that overlapped all groups, and the expressions of Wnt ligands such as Wnt3a, 4, and 5a were also confirmed (Figure 1(b)). Also, the relative level of CRP normalized by HSP70, which is an internal exosomal marker, was significantly increased in the TTX groups versus the control and 2 weeks in the NTX group (data not shown). To further investigate the expression of the CRP in total liver tissues and serum, we analyzed Western blot and ELISA. The protein level of CRP, as shown by ELISA and Western blot, was significantly upregulated by PD-MSC transplantation in the BDL rat model (^∗^^,#^*p* value ≤ 0.05) (Figures 1(c) and 1(d)). Thus, PD-MSC transplantation effectively induced CRP through exosome secretion in the BDL rat models.

### 3.3. The Expression of Wnt Signaling and Vascular Factors in BDL Rat Liver

**Figure 2:**
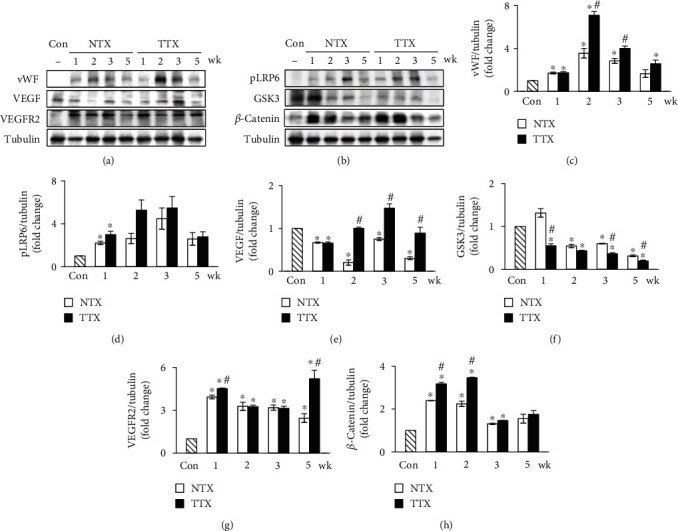
Induced Wnt signaling and angiogenesis by PD-MSC transplantation in BDL rats. Angiogenesis (a) and Wnt signaling (b) factors were detected in rat liver tissues after PD-MSC transplantation. Graph of intensity was normalized to that of tubulin for vWF (c), VEGF (e), VEGFR2 (g), pLRP (d), GKS3 (f), and *β*-catenin (h) (*n* = 2 per group, ^∗^Con and ^#^NTX vs. *p* < 0.05). Data were represented as the mean ± SD. vWF: von Willebrand factor; VEGF: vascular endothelial growth factor; VEGFR2: vascular endothelial growth factor receptor 2; Con: control group; NTX: PD-MSC nontransplanted group; TTX: PD-MSC transplanted group; wk: week.

To demonstrate the correlation between the Wnt pathway and the vascular formation following PD-MSC transplantation in liver cirrhosis, we analyzed the protein expression of factors related to angiogenesis and Wnt signaling in the BDL rat liver. The expression of vWF (von Willebrand Factor) and VEGF was significantly decreased in NTX groups versus control (^∗^*p* value ≤ 0.05) (Figures 2(a), 2(c), and 2(e)). The expression of VEGF was enhanced in the TTX groups compared to the NTX groups (Figures 2(a) and 2(e)) (^#^*p* value ≤ 0.05). In the case of VEGFR2, it was significantly increased in the TTX groups at 1 wk and 5 wks compared with the NTX groups (^#^*p* value ≤ 0.05) (Figures 2(a) and 2(g)). However, phospho-GSK3*αβ*, which is a *β*-catenin inhibitory molecule, were significantly downregulated while the angiogenic markers such as vWF, VEGF, and VEGFR2 were significantly increased in the liver tissues of the PD-MSC transplanted groups versus the control and NTX groups (^∗^^,#^*p* value ≤ 0.05) (Figures 2(b) and 2(f)). In particular, the expressions of the phosphorylated form of LRP and nonphosphorylated form of *β*-catenin, which are the receptor and key factor for the Wnt pathway, respectively, were promoted in the BDL-injured rat liver by PD-MSC transplantation (^∗^^,#^*p* value ≤ 0.05) (Figures 2(b), 2(d), and 2(h)). These findings indicate that PD-MSCs promote angiogenesis through activation of the Wnt pathway in chronic liver disease.

### 3.4. The Regulation of Angiogenesis and Wnt Signaling in WB-F344s by the CRP

**Figure 3:**
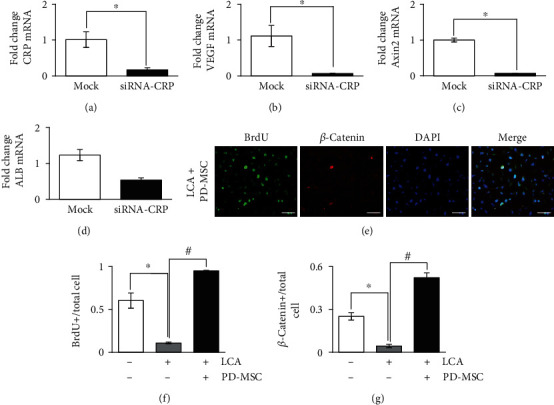
The direct effects of CRP in WB-F344s. mRNA levels of CRP (a), VEGF (b), Axin2 (c), and ALB (d) were evaluated in siRNA-CRP-transfected WB-F344s by qRT-PCR. Representative images of immunofluorescence were stained with WB-F344s for *β*-catenin and BrdU after LCA treatment and cocultivation of PD-MSCs (e). Scale bars: 50 *μ*m. BrdU- (f) and *β*-catenin- (g) positive cells were counted (*n* = 3 per group, ^∗^ control group and ^#^LCA-treated group vs. *p* < 0.05). Data were represented as the mean ± SD. ALB: albumin; LCA: lithocholic acid-treated group; PD-MSC: PD-MSC cocultivated group.

**Figure 4:**
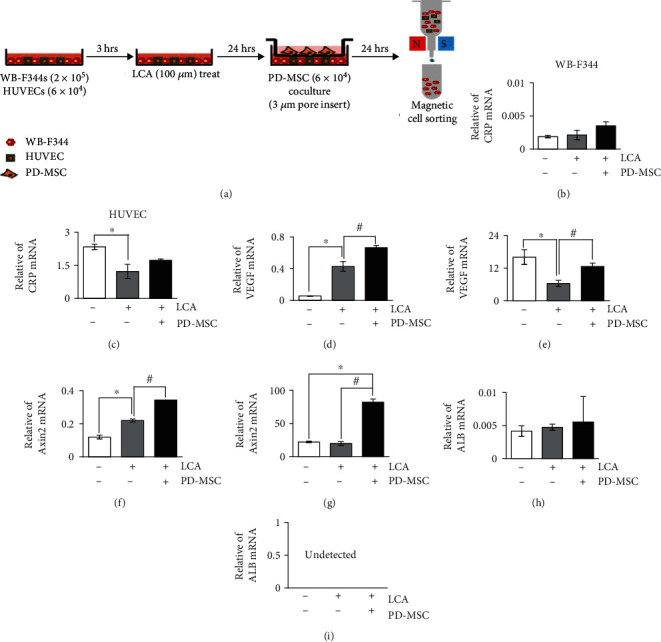
The effect of PD-MSC for WB-F344 and HUVEC in the mRNA level. The scheme for experimental protocol is shown in (a). The mRNA levels of CRP (b), VEGF (d), Axin2 (c), and ALB (d) were analyzed in WB-F344s treated with LCA and cocultivated with HUVECs and PD-MSCs. The mRNA levels of CRP (c), VEGF (e), Axin2 (g), and ALB (h) were detected in HUVECs treated with LCA and cocultivated with WB-F344s and PD-MSCs (*n* = 3 per group, ^∗^ control group and ^#^LCA-treated group vs. *p* < 0.05). Data were represented as the mean ± SD. ALB: albumin; LCA: lithocholic acid-treated group; PD-MSC: PD-MSC cocultivated group.

**Figure 5:**
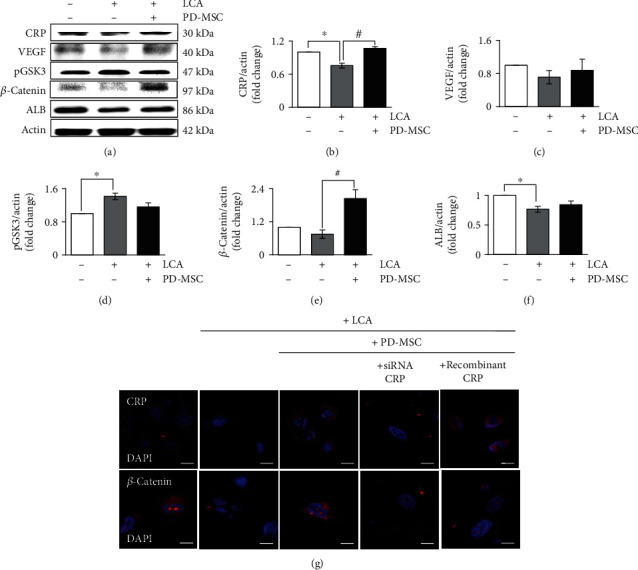
The effect of PD-MSC and CRP for WB-F344 in the protein level. The protein levels of CRP, VEGF, Wnt signaling factors, and ALB were detected in WB-F344s treated with LCA and cocultivated with HUVECs and PD-MSCs (a). Graph of intensity was normalized to that of actin for CRP (b), VEGF (c), pGKS3*αβ* (d), *β*-catenin (e), and ALB (f) (*n* = 2 per group, ^∗^ control group and ^#^LCA-treated group vs. *p* < 0.05). Data were represented as the mean ± SD. Representative images of immunofluorescence were stained with WB-F344s for CRP and *β*-catenin after LCA or recombinant CRP treatment, transfection of siRNA-CRP, and cocultivation of PD-MSCs (g). Scale bars: 20 *μ*m. ALB: albumin; LCA: lithocholic acid-treated group; PD-MSC: PD-MSC cocultivated group.

To investigate the direct functions of CRP related to vascular regeneration and the Wnt pathway in hepatocytes, we transfected siRNA-CRP in rat hepatocyte-like cells and analyzed the mRNA levels of CRP, VEGF, Axin2, and albumin (ALB). The expressions of CRP, VEGF, and Axin2 mRNA levels were significantly lower in the siRNA-CRP-transfected group than in the nontransfected group (mock) (^∗^*p* value ≤ 0.05) (Figures 3(a)–3(c)). Also, in siRNA-CRP-transfected rat hepatocyte-like cells, the expression of ALB, which is a hepatic function marker, had a tendency to decrease by silencing the CRP (Figure 3(d)). Most of all, in WB-F344s cocultivated with HUVECs directly and PD-MSCs indirectly as shown Figure 4(a), the expression of CRP and *β*-catenin was inhibited in the LCA treatment group. Also, their expressions were suppressed in the siRNA-CRP transfection group though cocultivation with PD-MSCs (Figure 5(g)). Therefore, the CRP directly modulates angiogenesis through the Wnt signaling and promotes hepatic regeneration.

### 3.5. The Expression of CRP, BrdU, and *β*-Catenin in WB-F344s Cocultivated with PD-MSCs

To determine the localization of *β*-catenin and proliferation of WB-F344s, we stained active *β*-catenin and BrdU and also investigated their localization in WB-F344s treated with LCA and cocultivated PD-MSCs through immunofluorescence. The BrdU and *β*-catenin were localized in the nucleus (Figure 3(e) and Supplement Figure 2). As the *β*-catenin- and BrdU-positive cells were counted, they were significantly increased in the group treated with LCA and cocultivated PD-MSCs compared to the non-cocultured group (^∗^^,#^*p* value ≤ 0.05) (Figures 3(f) and 3(g)). These results indicate that CRP increased by PD-MSCs in WB-F344s promotes the Wnt pathway and enhances hepatic regeneration.

### 3.6. The Correlation of CRP, Vascular Factors, and Wnt Signaling Factors in WB-F344s and Endothelial Cells Cocultivated with PD-MSCs

To demonstrate the interaction between hepatocyte-like cells and endothelial cells via CRP and the Wnt signaling pathway, we used the culture scheme shown in Figure 4(a). The expression mRNA level of CRP, VEGF, Axin2, and ALB was analyzed in WB-F344s and HUVECs. In sorted WB-F344s and HUVECs, the mRNA level of CRP was upregulated by PD-MSC cocultivation (Figures 4(b) and 4(c)). Additionally, the expression of VEGF and Axin2 was significantly increased in LCA-treated and PD-MSC-cocultivated WB-F344s and HUVECs compared to the LCA-treated group (Figures 4(d)–4(g)). In the WB-F344s, ALB, a hepatic epithelial marker, had an upregulated tendency in the PD-MSC-cocultivated group (Figure 4(h)). However, albumin was not detected in the sorted HUVECs (Figure 4(i)). Western blot analysis in WB-F344s showed that the protein expression of CRP is increased in the LCA-treated and PD-MSC-cocultured group (Figures 5(a) and 5(b)). Additionally, the expression pattern of factors related to the Wnt pathway (Figures 5(a), 5(d), and 5(e)), VEGF (Figures 5(a) and 5(c)), and ALB (Figures 5(a) and 5(f)) was matched with the pattern of CRP in that group. These results suggest that the CRP upregulated by PD-MSCs prompts vascular regeneration via Wnt signaling in WB-F344s by contacting HUVECs.

## 4. Discussion

MSCs show self-renewal and can differentiate into multiple cell lineages [27, 28]. In addition to these properties, homing, in which cells migrate to injured areas owing to chemical gradients which has been suggested as a therapeutic advantage, is a characteristic of MSCs [6, 29]. Nevertheless, stem cell therapy still has several disadvantages, such as poor isolation of the cells, efficiencies of migration and survival of the transplanted cells, and obscure mode of action [30].

Recently, the secretome, which comprises the secreted substances in cell-to-cell communication, has been used as a therapeutic target [31]. The secretome is known as the mass of molecules or factors released into the extracellular space; therefore, it can be utilized as a cell-free therapy [32]. The use of cell-free therapy through MSC-released secretomes in regenerative medicine has strengths compared to that of transplantation of stem cells. Utilization of the secretome solves several problems related to safety, such as tumorigenicity, immune compatibility, and infections. The application of secretomes released by MSCs, such as conditioned medium (CM), is more practical and economical than stem cell transplantation because the cell collection procedures can be avoided, and high levels of secretome production are possible [33–35]. Exosomes among the secretome have been considered a key therapeutic microvesicle because they secrete extracellular vesicle- (EV-) carrying enzymes or cellulases into the vessels [36]. Royo et al. demonstrated that exosomes can be used to quantify different chemical metabolites, including metabolites involved in arginine metabolism, which controls the function of vessels. In EV excreted from the serum *in vivo*, arginase activity was detected in the isolated EVs, and this vesicular activity, through Dil uptake, significantly increased underdamaged liver conditions. Finally, the researchers demonstrated that the exosomes secreted by hepatocytes are metabolically active; for example, they show oxidative stress metabolism and endothelial function [37]. Similarly, our study evaluated the expression of vWF, VEGF, and its receptors for endothelial function by exosomes excreted by rat serum (Figures 2(a), 2(c), 2(e), and 2(g)). Increased exosomes due to the interaction of WB-F344s, HUVECs, and PD-MSCs promoted angiogenesis and upregulated key factors related to vascularization, such as VEGF (Figures 4(d) and 4(e) and 5(a) and 5(c)). In another study, acetaminophen- or diclofenac-injured livers increased exosome release in hepatic cells as shown by ultra-high-performance liquid chromatography-mass spectrometry (UHPLC-MS). Then, the authors showed that the extracellular vesicles secreted by hepatic cells are metabolically active and function in the pathophysiological activity of several drugs in liver injury [14]. Also, Ferguson and his colleagues precipitated the miRNAs in MSC exosomes and performed a network analysis to confirm the principal biological pathways controlled by exosomes. In the present study, miRNA-targeted genes enriched for angiogenesis processes were associated with vascular regenerative influence. Our data also support that the CRP in exosomes secreted by PD-MSC transplantation is a trigger as a positive effector to regenerate damaged liver via activated angiogenesis in a rat model with hepatic failure. Targeted genes were related to Wnt and profibrotic signaling, apoptosis, and cell proliferation [38]. However, the mechanism of exosomes in hepatocytes was not elucidated in this study.

The exosomes released by MSCs as well as hepatocytes derived from many sites demonstrated the ability to regenerate hepatic tissue. Tingfen and colleagues transplanted exosomes excreted from human umbilical cord-MSCs (hucMSCs) to a CCl_4_-injured mouse model and found that surface fibrous capsules, hepatic inflammation, and collagen deposition were attenuated by exosomes released by hucMSCs. The exosomes derived from hucMSCs (hucMSC-Ex) also reduced the expression of transforming growth factor- (TGF-) *β*1 and phosphorylated Smad2 by suppressing epithelial-mesenchymal transition (EMT) [39]. In another study, exosomes released by adipose-derived MSCs (AD-MSCs) were tested for their effect on regeneration. Exosomes overexpressing miR181-5p in AD-MSCs inhibited Bcl-2 and Stat3 and promoted autophagy in hepatic stellate cells [40]. In addition, the exosomes isolated from bone marrow-derived MSCs (BM-MSCs) were assayed in hepatic disease models, such as CCl_4_-, hydrogen peroxide- (H_2_O_2_-), and acetaminophen- (APAP-) induced models. In these experiments, the exosomes led to the upregulation of proteins related to cell proliferation, such as PCNA, cyclin D1, and Bcl-xL in the exosome-treated group [41]. The effect of exosomes has not been related to hepatic regeneration in the BDL model although many reports have shown that exosomes have therapeutic abilities.

## 5. Conclusions

In conclusion, PD-MSC transplantation effectively increased CRP through exosome secretion in a BDL rat model. Additionally, CRP induced vascular formation via Wnt signaling and directly functions in hepatic regeneration of hepatocytes. These findings showed that PD-MSCs upregulate CRP and promote vascular regeneration through activation of the Wnt pathway in chronic liver cirrhosis. Our findings indicated that CRP in exosomes excreted by PD-MSCs has a role as a trigger factor for hepatic regeneration as well as angiogenesis via the Wnt signaling pathway. Therefore, these data could be useful to understand the role of CRP on therapeutic strategy in degenerative diseases.

## Data Availability

All data analyzed for this study are included in this article. Also, the data used to support the findings of this study are available from corresponding author upon request.

## Abbreviations

BDL: Bile duct ligation
BSA: Bovine serum albumin
CP-MSCs: Chorionic plate-derived MSCs
CRP: C-reactive protein
DAPI: 4′,6-Diamidino-2-phenylindole
DMEM/F12: Dulbecco's modified Eagle medium/Ham's F-12 medium
ELISA: Enzyme-linked immunosorbent assay
FATP: Fatty acid transport protein
FBS: Fetal bovine serum
HDL: High-density lipoprotein
hFGF-4: Human fibroblast growth factor-4
HO: Heme oxygenase
HRP: Horseradish peroxidase
LDL: Low-density lipoprotein
MSCs: Mesenchymal stem cells
PBS: Phosphate-buffered saline
PCR: Polymerase chain reaction
PD-MSCs: Placenta-derived MSCs
SDS-PAGE: Sodium dodecyl sulfate polyacrylamide gel electrophoresis
TBS: Tris-buffered saline.

## Supplementary Materials

Supplementary Materials Supplementary Figure 1: engraftment and tracing of PHK67 fluorescence-labeled human PD-MSC in BDL-injured rat livers. The engrafted PD-MSCs into the rat liver were detected and traced by labeling the PKH67 (green) using fluorescence microscope (a) and (c). *n* = 5 per group, scale bars: 20 *μ*m. Th expression of human-specific Alu sequence in BDL-injured rat livers after PD-MSC transplantation using quantitative real-time PCR analysis (b). *n* = 3 per group. ^∗^NTX group vs. *p* < 0.05. Data were represented as the mean ± SD. Supplementary Figure 2: the representative images of immunofluorescence were stained with WB-F344s for *β*-catenin and BrdU in normal and LCA-treated group. LCA: lithocholic acid-treated group. Scale bars: 50 *μ*m.
