# Supplementary material for: Exosomes from Placenta-Derived Mesenchymal Stem Cells Are Involved in Liver Regeneration in Hepatic Failure Induced by Bile Duct Ligation
Source: Stem Cells Int. 2020 Oct 9;2020:5485738. doi: 10.1155/2020/5485738 (PMC7568818; doi:10.1155/2020/5485738)
Supplement: Supplementary Materials — Supplementary Figure 1: engraftment and tracing of PHK67 fluorescence-labeled human PD-MSC in BDL-injured rat livers. The engrafted PD-MSCs into the rat liver were detected and traced by labeling the PKH67 (green) using fluorescence microscope (a) and (c). n = 5 per group, scale bars: 20 μm. Th expression of human-specific Alu sequence in BDL-injured rat livers after PD-MSC transplantation using quantitative real-time PCR analysis (b). n = 3 per group. ∗NTX group vs. p < 0.05. Data were represented as the mean ± SD. Supplementary Figure 2: the representative images of immunofluorescence were stained with WB-F344s for β-catenin and BrdU in normal and LCA-treated group. LCA: lithocholic acid-treated group. Scale bars: 50 μm. [file 5485738.f1.pdf]

Supplementary Materials

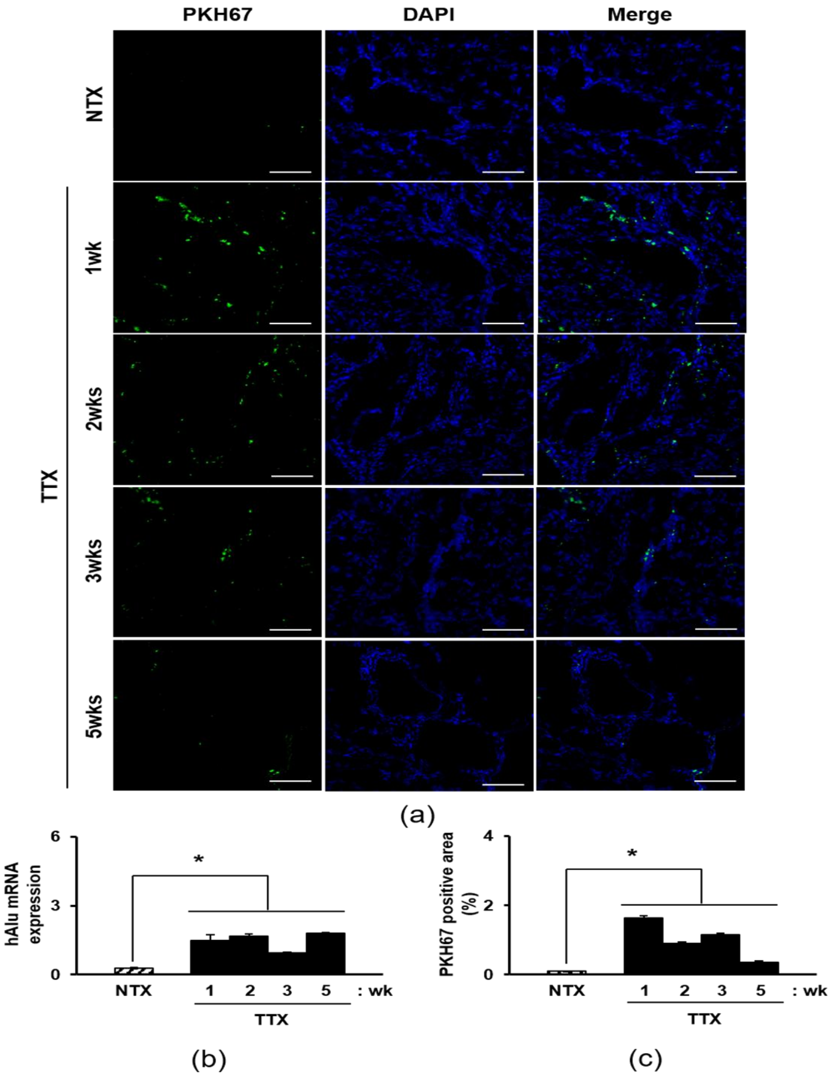

SUPPLEMENTARY FIGURE 1: Engraftment and tracing of PHK67 fluorescence labeled human PD-MSC in BDL-injured rat livers. The engrafted PD-MSCs into the rat liver were detected and traced by labeling the PKH67 (green) using fluorescence microscope (a) and (c). n=5 per group, Scale bars: 20μm. Th expression of human-specific Alu sequence in BDL-injured rat livers after PD-MSCs transplantation using quantitative real-time PCR analysis (b). n=3 per group. *, NTX group vs; $p<0.05$. Data were represented as the mean ± SD.

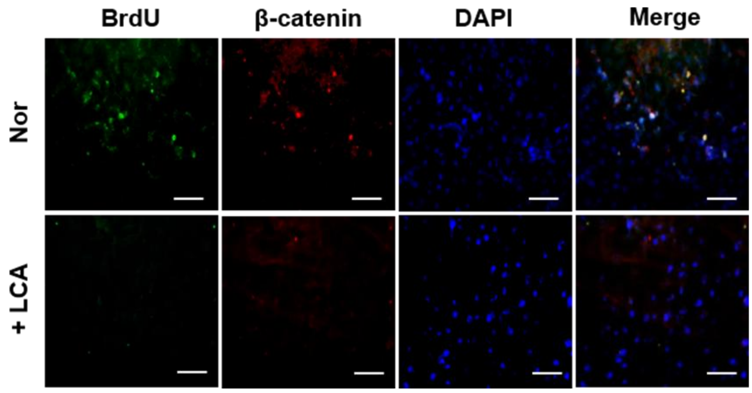

SUPPLEMENTARY FIGURE 2: The representative images of immunofluorescence were stained with WB-F344s for β-catenin and BrdU in normal and LCA treated group. LCA, lithocholic acid-treated group. Scale bars: 50μm.
